# Dr. Holmes on Physical Diagnosis (Extract)

**Published:** 1883-02

**Authors:** 


Dr. Holmes on Physical Diagnosis.—I have often felt, when seeing hospital patients worried by hammering and long listening to their breathing, in order that the physician might map out nicely the diseased territory, the boundaries of which he could not alter, as if it was too much like the indulgence of an idle, and worse than idle, curiosity. A confessor may ask too many questions; it may be feared that he has sometimes suggested to innocent young creatures what they would never have thought of otherwise. I even doubt whether it is always worth while to auscult and percuss a suspected patient. Nature is not unkind in concealing the fact of organic disease for a certain time. What is the great secret of the success of every form of quackery? *Hope kept alive.* What is the too fatal gift of science? *A prognosis of despair.* "Do not probe the wound too curiously," says Samuel Sharp, the famous surgeon of the last century. I believe a wise man sometimes carefully worries out the precise organic condition of a patient's chest when a *very* wise man would let it alone and treat the constitutional symptoms. The well-being of a patient may be endangered by the pedantic fooleries of a specialist.—*Boston Medical and Surgical Journal.*

